# Prevalence and symptoms of incidental meningiomas: a population-based study

**DOI:** 10.1007/s00701-025-06506-7

**Published:** 2025-04-03

**Authors:** Eddie de Dios, Olivia Näslund, Mansor Choudhry, Marcus Berglund, Thomas Skoglund, Darko Sarovic, Lina Rydén, Silke Kern, Ingmar Skoog, Erik Thurin

**Affiliations:** 1 Department of Radiology, Institute of Clinical Sciences, Sahlgrenska Academy, University of Gothenburg, Gothenburg, Sweden; 2 Department of Radiology, Sahlgrenska University Hospital, Region Västra Götaland, Gothenburg, Sweden; 3 Department of Clinical Neuroscience, Institute of Neuroscience and Physiology, University of Gothenburg, Gothenburg, Sweden; 4 Department of General Surgery, Sahlgrenska University Hospital, Region Västra Götaland, Gothenburg, Sweden; 5 Department of Neurosurgery, Sahlgrenska University Hospital, Region Västra Götaland, Gothenburg, Sweden; 6 Department of Radiology, Harvard Medical School, Cambridge, MA USA; 7 Athinoula a. Martinos Center for Biomedical Imaging, Department of Radiology, Massachusetts General Hospital, Boston, MA USA; 8 Department of Psychiatry and Neurochemistry, Institute of Neuroscience and Physiology, Sahlgrenska Academy, Center for Ageing and Health (AGECAP) at the University of Gothenburg, Mölndal, Sweden; 9 Department of Neuropsychiatry, Region Västra Götaland, Sahlgrenska University Hospital, Gothenburg, Sweden

**Keywords:** Asymptomatic meningioma, Incidental meningioma, Brain imaging, Cohort study, Incidence, Conservative treatment

## Abstract

**Background:**

Meningioma is the most common intracranial primary neoplasm and is often discovered accidentally. Common and non-specific symptoms such as headache and dizziness may be wrongfully attributed to meningiomas, which can lead to unnecessary surgery and anxiety for the patient. Understanding the prevalence of meningioma is therefore pivotal to assess the burden of this disease and determine indications for surgery.

**Method:**

Participants in this study were recruited through “The Gothenburg H70 Birth cohort study” wherein the health of 70-year-olds is examined. Clinical characteristics and symptoms such as sex, body mass index, history of smoking, previous head trauma, previous seizure, headache, dizziness, dementia, and life quality were evaluated. The associations between these variables and the presence of meningioma on MRI were determined.

**Results:**

MRI examinations were collected from 792 participants (415 [52.4%] women) in “The Gothenburg H70 Birth cohort study”. The prevalence of meningioma was 1.8% (*n* = 14). Meningiomas were more common in women (*n* = 12) than men, but no other significant differences were observed between participants with and without meningiomas.

**Conclusions:**

Meningiomas are common among older women, yet often asymptomatic. Caution should be exercised when attributing symptoms to incidentally discovered small meningiomas, and a conservative approach to treatment may be warranted in these cases.

## Introduction

Meningiomas are slow-growing extra-axial intracranial tumors originating from arachnoid cap cells [22]. They are the most common intracranial primary neoplasm, constituting around one third of all brain tumors reported to the Central Brain Tumor Registry of the United States (CBTRUS). Meningiomas are more common in women and their prevalence increases with age [2, 15, 16, 26]. Previous research has indicated that asymptomatic meningiomas are common among older adults. In the group aged 75 years and older, the incidence rate of meningiomas reported to the CBTRUS was 42 per 100 000 person years [15]. A smaller study has suggested a prevalence of 2.8% in asymptomatic 75-year-old women when examined with MRI [11]. In concordance, an autopsy study reported a prevalence of undiagnosed asymptomatic meningiomas of 2–3% [10], whereas the 2007 Rotterdam study demonstrated a prevalence of asymptomatic meningiomas of 1.0% in the age group 60–74 years [24]. Asymptomatic meningioma rates thus seem to be related to the number of patients undergoing brain computed tomography (CT) or magnetic resonance imaging (MRI), indicating that the prevalence of asymptomatic meningiomas is likely underrated [6]. This was later confirmed in a 2014 Norwegian study, demonstrating a linear relationship between the number of MRI scans performed and the number of extra-axial (but not intra-axial) brain tumors diagnosed [23]. The difference between intra- and extra-axial tumors was suggested to result from intra-axial tumors (like gliomas) eventually causing symptoms, while extra-axial tumors (like meningiomas) are more likely to remain asymptomatic throughout life.

As meningiomas are often discovered accidentally in the setting of non-specific symptoms, such as headache or fatigue, it is sometimes uncertain if there is a causal relationship between these symptoms and the discovered meningioma. Although current guidelines state that a wait-and-scan approach is preferrable when the patient is asymptomatic [5], symptoms may wrongfully be attributed to patients, especially concerning vague and non-specific ones such as headache and fatigue. To minimize the risk of unnecessary surgery, it is therefore vital to determine the prevalence of meningiomas in the elderly population and to determine if incidental meningiomas are associated with symptoms such as headache and dizziness.

To avoid sampling bias associated with hospital care, and confounders related to the decision to perform an MRI examination, we used a random population sample. To our knowledge, the relationship between meningiomas and various clinical variables has not been thoroughly examined using population-based data before. We therefore conducted a representative population-based study to investigate the prevalence of meningiomas and their association with neurological symptoms in individuals aged 70 years.

## Methods

This study was conducted according to the Helsinki Declaration and approved by the Regional Ethical Review Board in Gothenburg (approval numbers: 869–13, T076-14, T166-14, 976–13, 127–14, T936-15, 006–14, T703-14, 006–14, T201-17, T915-14, 959–15, T139-15), and by the Radiation Protection Committee (approval number: 13–64). Study participants provided written informed consent prior to any examinations related to the study. If a participant was unable to provide consent, for instance due to dementia, consent was obtained from a close relative.

### Study population

**Fig. 1:**
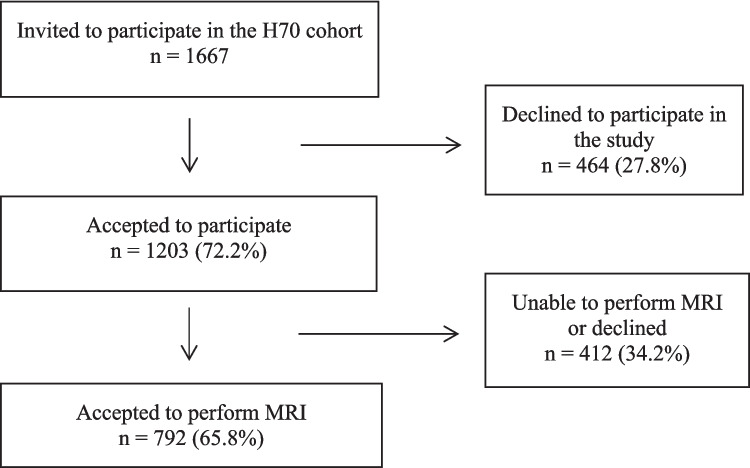
Flow-chart of patient selection

The subjects of this study were participants in “The Gothenburg H70 Birth cohort study” hence forth called “the H70 study” (https://www.gu.se/en/research/the-gothenburg-h70-birth-cohort-study), a longitudinal population-based study on the health of 70-year-old persons in the Gothenburg area in Sweden [19]. A summary of the inclusion and exclusion process is outlined in Fig. 1. The cohort was collected between 2014–2016 and all men and women that were registered as residents in Gothenburg and born on specific dates in 1944 were eligible for inclusion. A total of 1203 participants were included in the initial examination (response rate 72.2%). All participants were invited to also undergo brain imaging with both CT and MRI [19]. MRI was performed on 792 individuals, all of whom were included as the final patient cohort in the present study.

### Data collection

Demographic and clinical data were collected through the H70 study framework, which collects information on health-related aspects of life including socioeconomic factors, physical and mental health, functional and cognitive ability, and protective and risk factors of disease. Semi-structured interviews were also performed with a close informant, including questions about changes in intellectual function and behavior.

Collected data for the present study included sex (female/male), body mass index (BMI), diagnosis of dementia (yes/no), as well as interview answers concerning smoking history (yes/no), any previous severe head trauma (yes/no), suffering from headaches (yes/no), suffering from dizziness (yes/no), and any previous seizure (yes/no). Respondents were also asked to perform a self-assessment of Quality of Life (QoL) rated on a scale from 1 to 7, with 1 meaning “completely dissatisfied” and 7 meaning “completely satisfied” [1].

Body mass index (BMI) was calculated as *weight / height*^*2*^. The cognitive and psychiatric evaluations in this study were part of a larger battery of semi-structured interviews performed as part of the H70 study. The tests were performed by a medical doctor, psychiatrist, or psychiatric research nurse and included several questionnaires and personality tests, as outlined by Rydberg et al. [19]. Dementia was diagnosed according to the DSM-III R criteria (except that short- *or* long-term memory impairment was sufficient to fulfill the criteria in the H70 study), based on the neuropsychiatric examination and a close-informant interview, as described in detail previously [25].

### MRI acquisition and assessment

MRI examinations were acquired using a 3-Tesla MR scanner and sequences used for this study were a T1-weighted 3D sequence with an isotropic voxel size of 1 mm^3^, an axial T2-weighted 2D sequence with a slice thickness of 4 mm, and a sagittal fluid attenuation inversion recovery sequence with a slice thickness of 2 mm. Images were clinically assessed by general radiologists or neuroradiologists at a private radiology department. To include also small incidental meningiomas, an additional second reading was performed on all exams focused only on meningioma findings using T1-weighted images. All findings indicating meningioma were also evaluated by an experienced neuroradiologist in a consensus reading.

### Statistical analyses

Categorical variables are reported with frequency and percentage, and continuous variables with mean and standard deviation (SD). Comparisons were made between participants with and without meningioma using Fisher’s exact test for categorical variables and independent t-tests for continuous variables. Two-tailed p-values were used with statistical significance set to p ≤ 0.05. All statistical analyses were performed with SPSS, version 29.

## Results

Among 792 included participants (52.4% female), meningioma was present in 14 (1.8%), of whom 12 (85.7%) were females. Baseline characteristics are presented in Table 1, and comparisons between meningioma participants and non-meningioma participants are presented in Table 2. Female sex was associated with an increased probability of meningioma (*p* = 0.01). BMI, previous smoking, previous head trauma, dementia, headache, dizziness, previous seizure, and QoL were not significantly associated with meningioma.

**Table 1:** Baseline characteristics of the 792 participants included through the Gothenburg H70 Birth cohort study

Variable, unit	H70 cohort	Missing entries
Female, n (%)	415 (52.4%)	0
BMI, kg/m^2^ ± SD	26.0 ± 4.3	3 (0.4%)
Never smoked, n (%)	305 (38.5%)	0
Previous head trauma, n (%)	255 (32.2%)	0
Dementia, n (%)	13 (1.6%)	0
Headache, n (%)	209 (26.4%)	0
Dizziness, n (%)	164 (20.8%)	2 (0.3%)
Previous seizure, n (%)	25 (3.2%)	3 (0.4%)
QoL, mean ± SD	5.99 ± 1.22	25 (3.2%)

*BMI* body mass index, *QoL* Quality of Life, *SD* standard deviation

**Table 2:** Characteristics of participants with and without meningioma, including two-tailed p-values for comparisons between the groups

Variable, unit	Meningioma group (*n *= 14)	Non-meningioma group (*n* = 778)	*p*-value
Female sex, n (%)	12 (85.7%)	403 (51.8%)	**0.01**
BMI, kg/m^2^ ± SD	27.2 ± 3.0	25.9 ± 4.3	0.28
Never smoked, n (%)	4 (28.6%)	301 (38.7%)	0.44
Previous head trauma, n (%)	5 (35.7%)	250 (32.1%)	0.78
Dementia, n (%)	1 (7.1%)	12 (1.5%)	0.10
Headache, n (%)	5 (35.7%)	204 (26.2%)	0.42
Dizziness, n (%)	3 (21.4%)	161 (20.7%)	0.95
Previous seizure, n (%)	1 (7.1%)	24 (3.1%)	0.39
QoL, mean ± SD	6.17 ± 1.85	5.99 ± 1.21	0.62

*BMI* body mass index, *QoL* Quality of Life, *SD* standard deviation

## Discussion

In this study, the prevalence of meningioma was 1.8%. In a recent study, the prevalence of meningiomas was somewhat lower at 0.7%, despite a higher proportion of women (57%). However, this difference could be explained by the generally younger age of that cohort, with a mean age of 55 ± 14 years [12]. Our finding is instead more on par with the Rotterdam study, which had an identical proportion of women (52.4%) and reported a prevalence of 1.0% and 1.6% in the age groups of 60–74 years and 75–97 years, respectively [24]. In the Rotterdam study all MRIs were performed with a 1.5-Tesla scanner, which has a lower signal to noise ratio. Also, no dedicated meningioma-specific second assessment was performed. These circumstances could partly account for the slight difference in prevalence, but other factors including demographics and differences in the definition of meningioma are also possible.

The association between female sex and a higher prevalence of meningioma was confirmed, but no other statistically significant associations were observed between participants with and without meningioma. This important finding challenges the assumption that commonly reported symptoms, such as headache and dizziness, as well as QoL disturbances, are directly attributable to meningioma. Not only does our study suggest that these symptoms are not more prevalent in the meningioma group, but it also highlights the importance of considering alternative explanations for these symptoms in affected patients. Furthermore, caution should be exercised when using these symptoms as a basis for surgical decision-making, unless there is clear evidence that the characteristics of the meningioma, such as its location or contribution to increased intracranial pressure, are the underlying cause.

It is well-known that incidental imaging findings can lead to significant psychosocial distress. In a survey with 394 participants, 28.6% reported moderate to severe psychological distress after being notified of an incidental finding following whole-body MRI. The study also demonstrated a strong disagreement between the subjective and the radiological evaluation regarding the severity of the findings [21]. Therefore, it is not only important to avoid unnecessary surgery, but also to properly inform the patient that it is unlikely that a small, incidentally discovered meningioma is the cause of non-specific symptoms. This approach can lead to reduced anxiety for the patient, fewer unnecessary follow-ups, and easier management for the clinician.

Despite the often small size and indolent behavior of incidental meningiomas during follow-up [6], a systematic review evaluating managing strategies for incidentally diagnosed meningiomas found that surgery was performed in 27.3%, stereotactic radiosurgery in 22%, and active monitoring in 50.7%. They also concluded that there is a lack of consensus on how meningioma growth should be defined [7]. Previous research on growth patterns have indicated absence of calcification, T2-weighted hyperintensity, large tumor volume, and peritumoral edema as predictors of meningioma growth [8, 13, 14, 18, 27], whereas clinical features such as sex and use of estrogen-based hormone replacement therapy have shown mixed results [2, 4, 9, 17]. Furthermore, it is important to correlate meningioma growth with symptoms. A 5-year prospective study with 64 patients showed that although 75% of incidental meningiomas increased in volume, none of the patients developed tumor-related symptoms. Among the tumors that grew, 60% displayed a self-limiting growth pattern, concluding that serial imaging until symptomatic and/or persistent radiological growth is a safe management strategy in this patient group [3]. This suggests that incidental meningiomas are not only asymptomatic at diagnosis, but that they also tend to remain asymptomatic despite that the majority will grow during a 5-year period.

Strengths of this study are the large sample size of participants aged 70 years and the uniform assessment of high-resolution MRI sequences. The second screening dedicated to meningioma detection and subsequent corroboration by an experienced neuroradiologist further increases the diagnostic accuracy and the validity of our findings. Missing data were kept to a minimum through the H70 study and except for the 3.2% missing QoL entries, the other variables had no more than 0.4% missing entries among those who accepted to participate in the study. Through this random population sample, concerns regarding sampling bias associated with hospital care or the decision to perform an MRI examination were minimized. Furthermore, as the participants reported symptoms independent of the potential imaging findings, and the radiologists were blinded to this information, it is reasonable to assume that classification bias is also minimal.

A limitation of this study is the cross-sectional design, limiting the ability to draw conclusions regarding causality. Another limitation is the potential for selection bias as roughly half of the invited subjects either declined to participate in the study or were unable or unwilling to undergo the MRI examination. This could somewhat increase the risk of type 2-errors although none of the symptom variables demonstrated any tendency towards a significant association with a meningioma finding. Furthermore, a non-response analysis showed that there were no differences regarding sex and BMI between participants who underwent the MRI examination and those who did not. There were slightly more participants who were current smokers or had dementia that did not undergo the MRI examination [20]. However, associations between these characteristics and meningioma were not found in our study.

Further limitations were the small number of patients with meningioma in the cohort and the lack of further clinical information, such as size, localization, number of meningiomas in the same participant, previous skull surgery, or previous radiotherapy.

In conclusion, this population-based study confirms that meningiomas are relatively common among older women, yet often asymptomatic. Our results suggest that caution should be exercised when attributing symptoms to incidental meningiomas.

## Data Availability

No datasets were generated or analysed during the current study.

## Abbreviations

BMI: Body Mass Index
CT: Computed Tomography
CBTRUS: Central Brain Tumor Registry of the United States
H70: The Gothenburg H70 Birth cohort study
MRI: Magnetic Resonance Imaging
QoL: Quality of Life
SD: Standard Deviation
